# Prophylactic use amphotericin B use in patients with hematologic disorders complicated by neutropenia: a systematic review and meta-analysis

**DOI:** 10.1038/s41598-023-41268-1

**Published:** 2023-08-27

**Authors:** Zhaoyan Chen, Qiyi Feng, Xiaoxing Wang, Fangyuan Tian

**Affiliations:** 1 Department of Pharmacy, West China Hospital, Sichuan University, Chengdu, 610041 Sichuan People’s Republic of China; 2 Sichuan Provincial Key Laboratory of Precision Medicine and National Clinical Research Center for Geriatrics, Precision Medicine Research Center, West China Hospital, Sichuan University, Chengdu, 610041 Sichuan People’s Republic of China; 3 Department of Pharmacy, China-Japan Friendship Hospital, Beijing, 100029 China

**Keywords:** Cancer, Infectious diseases

## Abstract

The purpose of this study is to evaluate the efficacy of prophylactic use amphotericin B in patients with hematologic disorders complicated by neutropenia. We searched the PubMed, EMBASE, The Cochrane Library, CBM, CNKI, VIP and WanFang Data database and the China Clinical Trials Registry (www.chictr.org.cn) to collect randomized controlled trials (RCTs) of amphotericin B for patients with hematologic disorders complicated by neutropenia from inception to May 2023. The Cochrane risk-of-bias tool for RCTs was used to assess the bias risk of the included studies. The meta-analysis was performed using RevMan 5.3 software. A total of 6 studies with a total of 1019 patients were included. The results of the meta-analysis showed that the treatment group was superior to the control group in terms of the fungal infection rate, and the differences were statistically significant [RR = 0.47, 95% CI (0.32, 0.69), *P* < 0.0001]. There was no significant difference between the two groups in terms of the mortality [RR = 0.87, 95% CI (0.61, 1.23), *P* = 0.43] and the incidence of colonization [OR = 0.51, 95% CI (0.25, 1.03), *P* = 0.06]. The evidence shows that amphotericin B prophylactic use for patients with hematologic disorders complicated by neutropenia can decrease the fungal infection rate. However, there was no significant difference in reducing mortality or the incidence of colonization. Due to the limited quality and quantity of the included studies, more high-quality studies are needed to verify the above conclusion.

## Introduction

Patients with hematological malignancy who were treated with long-term chemotherapy in combination with broad-spectrum antibiotics and immunosuppressant therapy were often immunocompromised. In addition, due to the associated neutropenia caused by intensive chemotherapy, patients with hematologic malignancies have an increased risk of fungal infections. As it may be difficult to diagnose at the early stage and hard to cure, mortality rates exceed 50% in patients with fungal infection especially invasive fungal infection^1,2^.

Amphotericin B (Am B), as one of the commonly used polyene antifungal antibiotics, can damage membrane permeability by binding to ergosterol on the cell membrane, resulting in metabolic destruction and cell lysis, which acts as the first-line treatment for invasive fungal infection^3,4^. Recently, strategies of preventive therapy in susceptible populations have focused on using antifungal antibiotics to reduce the risk and improve the prognosis of fungal infection. The results of a study of people at high risk of opportunistic infection with prophylactic antimicrobial treatment have been promising^5^.

To date, no systematic review to our knowledge has reported the efficacy profile of Am B prophylactic use on patients with hematologic disorders complicated by neutropenia based on high-quality RCTs. Based on this, we performed a meta‐analysis of randomized controlled trials (RCTs) around the world to compare and analyze the efficacy of Am B on patients with hematologic disorders complicated by neutropenia^6–11^.

## Methods

### Eligibility criteria

Inclusion criteria: required for searching included the following: human studies; hematologic disorders population (age ≥ 18) complicated by neutropenia (neutrophil counts ≤ 500/mm^3^) duration ≥ 10 days, published RCT studies determining the efficacy of the Am B prophylactic use. Experimental: administration of Am B by intravenous injection or aerosol inhalation; Control: placebo or no treatment. Aerosol inhalation is administered by jet or ultrasonic nebulizer, and the dose is adjusted according to creatinine clearance. The other symptomatic treatments in the two groups were the same, and the duration of administration was not limited. Furthermore, the exclusion criteria were as follows: studies on animals or in vitro/ex vivo; adult population (aged < 18 years); published clinical studies without the full text or without original data, including reviews, editorials, case reports, and comments; and ongoing clinical studies whose basic information or protocol was unavailable. Major outcomes: 1) mortality including drug-associated mortality, infection-associated mortality, defined as the number of deaths/total number × 100%, drug-associated mortality defined as death due to adverse drug reactions, infection-associated mortality defined as death due to infection; 2) fungal infection rate: defines as the symptomatic presence of fungi at a superficial site, such as skin, without evidence of tissue invasion; 3) incidence of colonization: refers to a large number of spore growing fungi in the parts of the human body connected to the outside world, such as the digestive tract, upper respiratory tract, urogenital tract, etc., without damage to local tissues or symptoms. The following data were extracted from the literature: basic information of the included studies, baseline characteristics of the included subjects, information on intervention measures, and outcome indicators.

### Search strategy

We followed PRISMA guidelines to conduct this systematic review. Medical subject headings and free words such as “Amphotericin B”, “Neoplasms”, “Neoplasms”, “Prophylactic”, and the following databases were searched for relevant studies: the Cochrane Library (2023 May 5th), PubMed, and EMbase. The China Biomedical Literature Database (CBM), China National Knowledge Infrastructure (CNKI), VIP and WanFang Data were searched until May 2023. Taking PubMed as an example, the search strategy was shown in Table 1.

**Table 1:** Search strategy in PubMed.

Number	Search strategy
#1	Amphotericin B [MeSH Terms]
#2	Amphotericin [Text Word]
#3	Amphotericin B Cholesterol Dispersion [Text Word]
#4	#1 OR #2 OR #3
#5	Prophylactic[Text Word]
#6	Empirical[Text Word]
#7	#5 OR #6
#8	Neutropenia [MeSH Terms]
#9	Neutropenias [Text Word]
#10	#8 OR #9
#11	Neoplasms [MeSH Terms]
#12	Neoplasms [Title/abstract]
#13	#11 OR #12
#14	Randomized controlled trial
#15	Randomized controlled trial as topic
#16	Controlled clinical trial
#17	Controlled clinical trial as topic
#18	Random*
#19	#14 OR #15 OR #16 OR #17 OR #18
#20	#4 AND #7 AND #10 AND #13 AND #19

### Literature screening

To ascertain whether each study complied with the predetermined inclusion criteria, two reviewers (FY Tian and ZY Chen) independently read the titles and abstracts of the studies. To determine whether further review was necessary, all titles and abstracts were evaluated. The first 50 references were separately evaluated for quality control by a senior researcher. The degree of agreement was 90%, with five inconsistencies that were discussed among the three reviewers to reach agreement. The two reviewer groups then conducted a second round of review on the remaining studies. The references of the retrieved articles were further searched in an effort to locate more appropriate articles.

### Risk of bias assessment of included studies

The Cochrane Manual for Systematic Reviewers 5.1.0 was used in this study to assess the quality of enrolled RCTs and the risk of bias for seven domains as follows: sequence generation of random numbers, allocation concealment, blinding of participants, study personnel and outcome assessors, avoidance of incomplete outcome data or selective outcome reporting and discussion of other potential sources of bias.

### Statistical analysis

RevMan 5.3 software was used to conduct the statistical analyses. The χ2 test was used to assess heterogeneity among studies with the significance level α = 0.1. The proportion of statistical heterogeneity was assessed by the I^2^ measure. The random effects model was applied when the data were heterogeneous (I^2^ < 50%), and the fixed effect model was applied when the data were homogenous (I^2^ > 50%). The risk ratio (RR) was used for categorical variables to represent the effect size, and the interval was estimated using the 95% confidence interval (95% CI). Significant clinical heterogeneity was handled by subgroup analysis. A sensitivity analysis of the study was performed to examine the robustness of the results. Lastly, a funnel plot were used to assess publication bias.

## Results

### Data sources and searches

**Figure 1:**
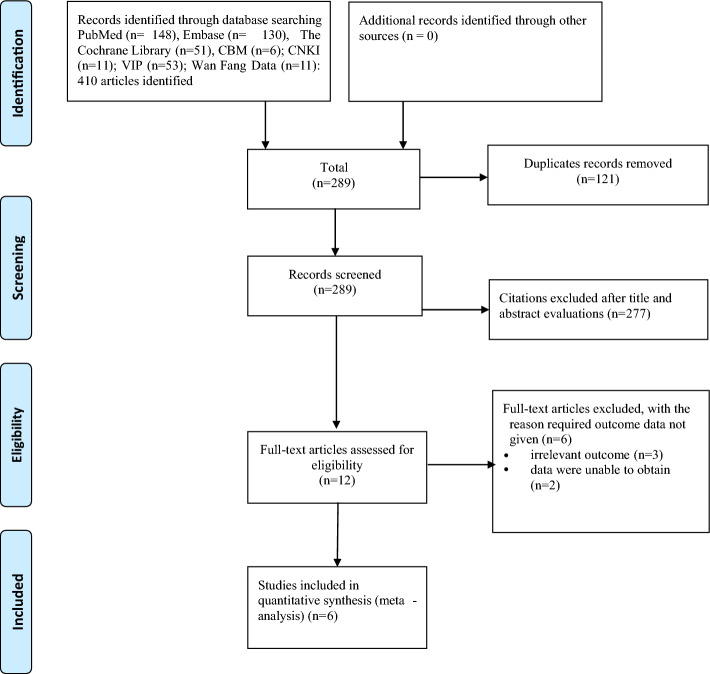
Flow diagram of include studies.

Duplicate articles were excluded, leaving 319 articles, and a total of 6 studies^6–11^ met the inclusion criteria and were ultimately included in our analysis (Fig. 1).

### Study selection and characteristics

**Figure 2:**
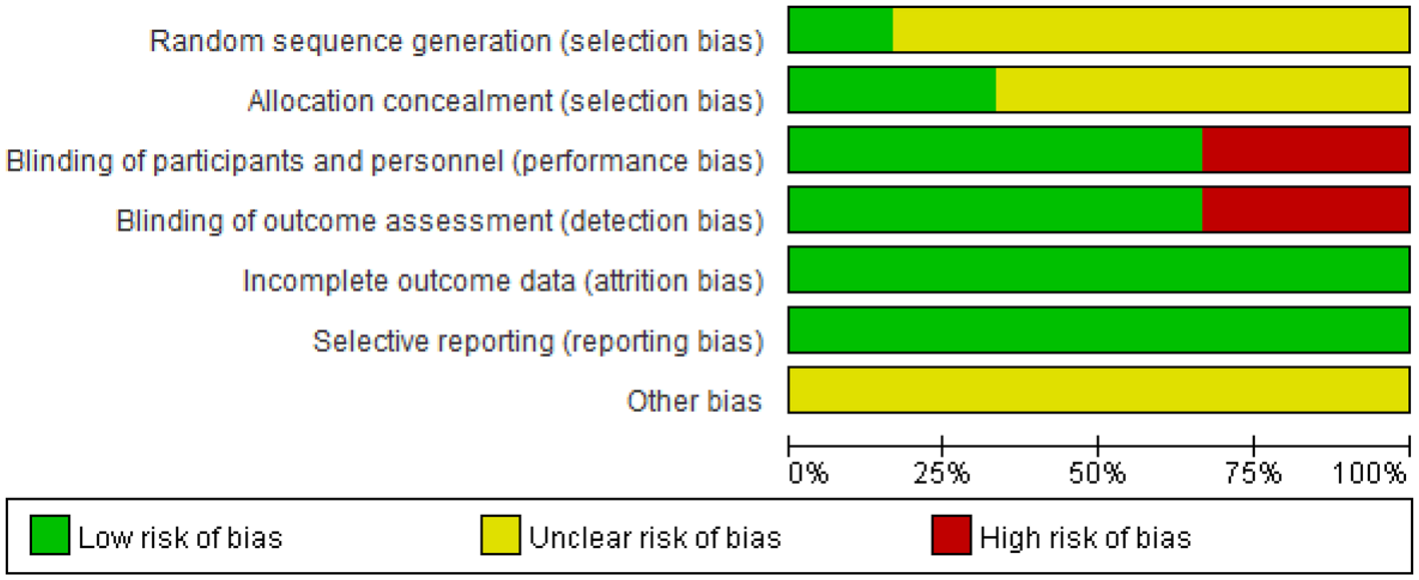
The percentage of risk of bias items of included studies.

A total of 1019 patients with hematological diseases complicated by neutropenia were included in 6 studies^6–11^, including 557 in the experimental group and 462 in the control group. All studies reported comparable baselines, and all studies reported fungal infection rates and mortality. The basic characteristics of the included studies are shown in Table 2, and the results of the risk of bias assessment of the included studies are shown in Fig. 2.

**Table 2:** General information of the included studies.

Study, year	Country/region	Sample size (T/C)	Age (T/C)	Gender (male)%		Classification of primary disease %		Medication purpose	Interventions		Outcomes
				T	C	T	C		T	C	
Rijnders 2008^6^	Netherlands	139/132	49(18–73)/50(20–74)	55.4	61.4	AML/MDS: 47%	AML-MDS: 51%	Prophylactic	Am B, 2.5 ml(5 mg/ml), biw, inh	Placebo	①②
						other: 53%	other: 49%				
Schwartz 1999^7^	Germany	227/155	46(16–80)/48(17–81)	NA	NA	AML/MDS/CML:73%	AML/MDS/CML:76%	Prophylactic	Am B, 5 ml(10 mg), bid, inh	NT	①②
						ALL/NHL: 10%	ALL/NHL: 8%				
						AutoBMT: 17%	AutoBMT: 16%				
Behre 1995^8^	Germany	65/50	43(19–73 43(18–81)	NA	NA	AML:58%	AML:58%	Prophylactic	Am B, 5 ml(10 mg), bid, inh	NT	①②
						ALL/NHL: 8%	ALL/NHL: 12%				
						Other: 34%	Other: 30%				
Riley 1994^9^	The United States	17/18	38(10–51)/38 (14–52)	41	33	AML/ALL/MDS/MM:59%	AML/ALL/MDS/MM:61%	Prophylactic	Am B, 0.1 mg/kg, qd, iv	Placebo	①②③
						Other: 41%	Other: 39%				
Perfect 1992^10^	The United States	91/91	37.9 ± 7.1/39.6 ± 7.7	12	18	AML/MM:11%	AML/MM:11%	Prophylactic	Am B, 0.1 mg/kg, qd, iv	Placebo	①②③
						Other: 89%	Other: 89%				
Pizzo1982^11^	The United States	18/16	18(8–30)/16(2–25)	78	50	AML/ALL: 67%	AML/ALL: 69%	Empirical	Am B, 0.5 mg/kg, qd, iv	Placebo	① ②
						Other: 33%	Other: 31%				

T Treatment group, C Control group, Am B Amphotericin b, NT No treatment, AML Acute myelocytic leukemia, ALL Acute lymphoblastic leukemia, MDS Myelodysplastic syndrome, BMT Bone marrow transplantation, NHL Non-Hodgkin lymphoma, NR Not reported, inh Inhalation. Outcomes: ① mortality, ② fungal infection rate, ③ incidence of colonization.

### Meta-analysis

#### Fungal infection rate of Am B versus placebo/NT

**Figure 3:**
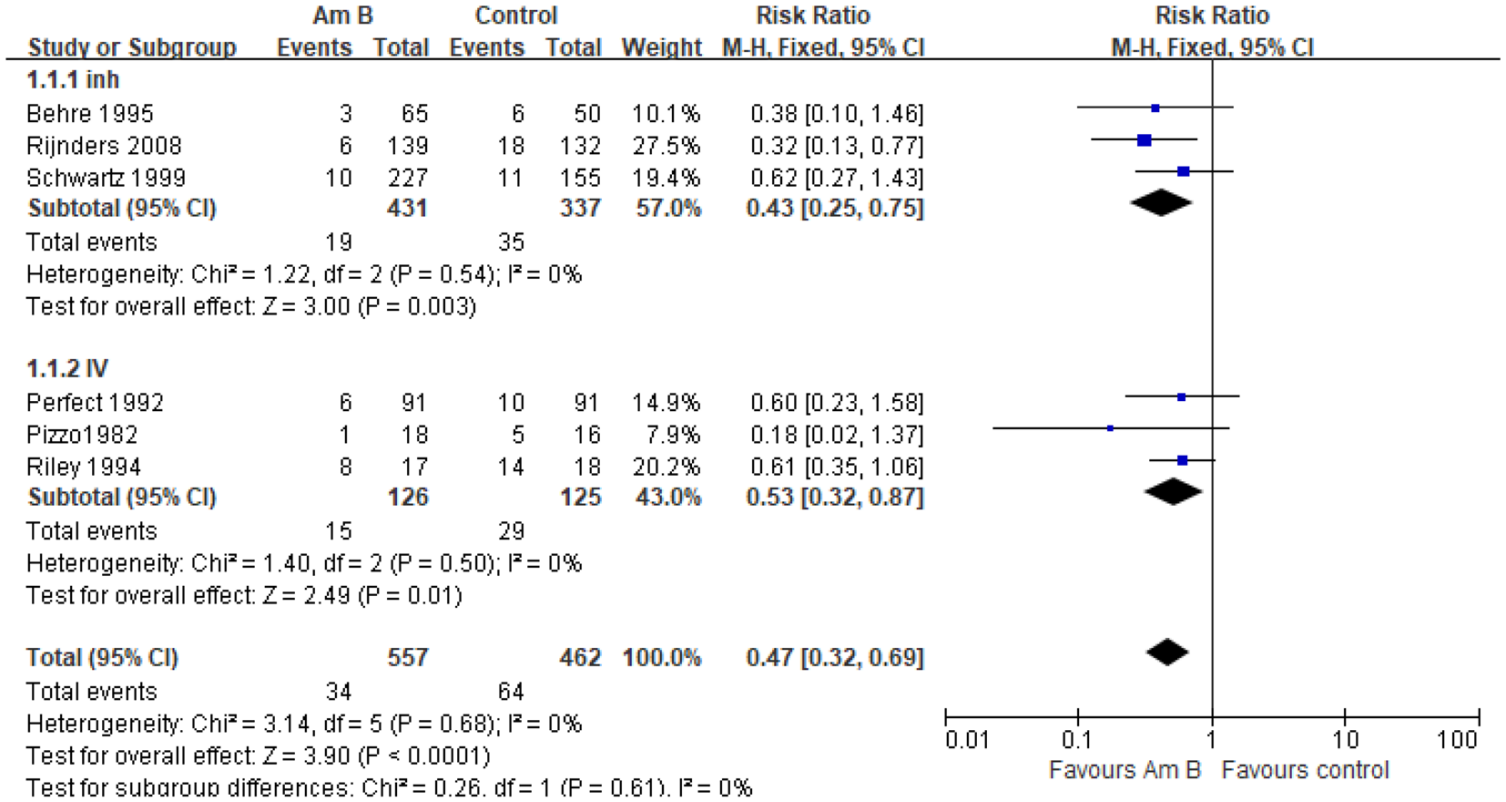
Forest plot of meta-analysis of fungal infection rate.

Six studies reported the fungal infection rate of prophylactic use of amphotericin B in patients with hematological disease complicated by neutropenia^6–11^. The meta-analysis of the fixed effect model (I^2^ = 0%, *P* = 0.68) showed that compared with the placebo or no-intervention group, the fungal infection rate in the experimental group was significantly decreased [RR = 0.47, 95% CI (0.32, 0.69), *P* < 0.0001]. Subgroup analysis showed that there was significant difference between the two groups with inh [RR = 0.43, 95% CI (0.25, 0.75), *P* = 0.003] or IV [RR = 0.53, 95% CI (0.32, 0.87), *P* = 0.01] (Fig. 3).

#### Mortality Am B versus Placebo/NT

**Figure 4:**
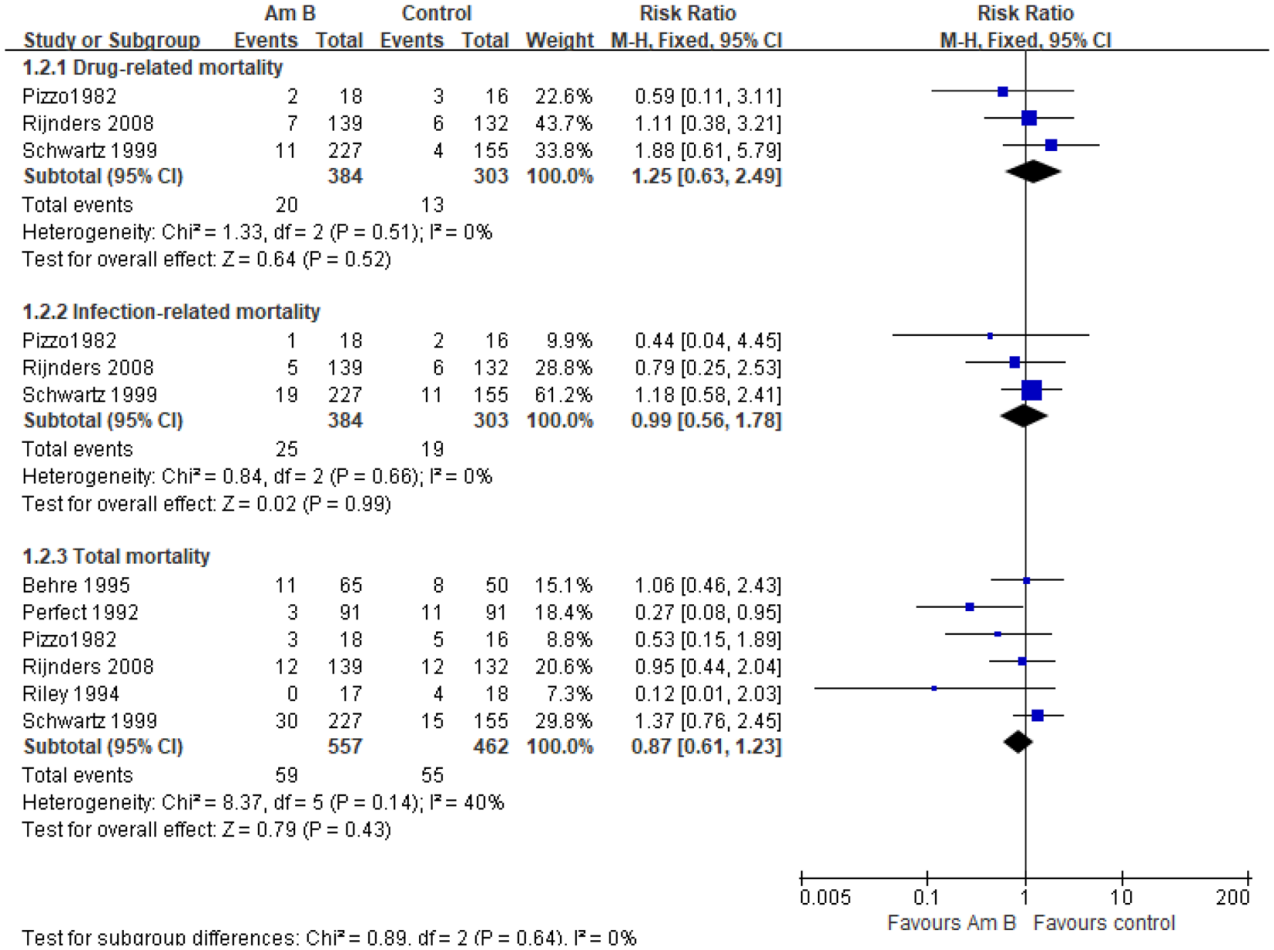
Forest plot of the meta-analysis of the mortality.

Six studies reported the overall mortality rate of prophylactic use of amphotericin B in patients with hematological complications of neutropenia^6–11^. The meta-analysis of the fixed effect model (I^2^ = 40%, *P* = 0.14) showed that compared with the placebo or no-intervention group, the overall mortality of patients in the experimental group was slightly reduced, but there was no significant difference among groups. [RR = 0.87, 95% CI (0.61, 1.26), *P* = 1.23]. Subgroup analysis showed that there was no significant difference between the two groups in drug-associated mortality [RR = 1.25, 95% CI (0.63, 2.49), *P* = 0.52] or infection-associated mortality [RR = 0.99, 95% CI (0.56, 1.78), *P* = 0.99] (Fig. 4). On sensitivity analysis, after removing each study by there was no significant difference between the two groups of mortality, suggesting that these results are relatively stable.

#### Incidence of colonization

**Figure 5:**
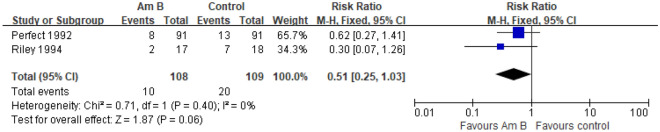
Forest plot of the meta-analysis of incidence of colonization.

Two studies reported the incidence of colonization of prophylactic use of amphotericin B in patients with hematological disease complicated by neutropenia^9,10^. The meta-analysis of the fixed effect model (I^2^ = 0%, *P* = 0.40) showed that compared with the placebo or no intervention group, the incidence of colonization in the experimental group was slightly lower, but there was no significant difference among groups [RR = 0.51, 95% CI] (0.25, 1.03), *P* = 0.06] (Fig. 5).

#### Publication bias

**Figure 6:**
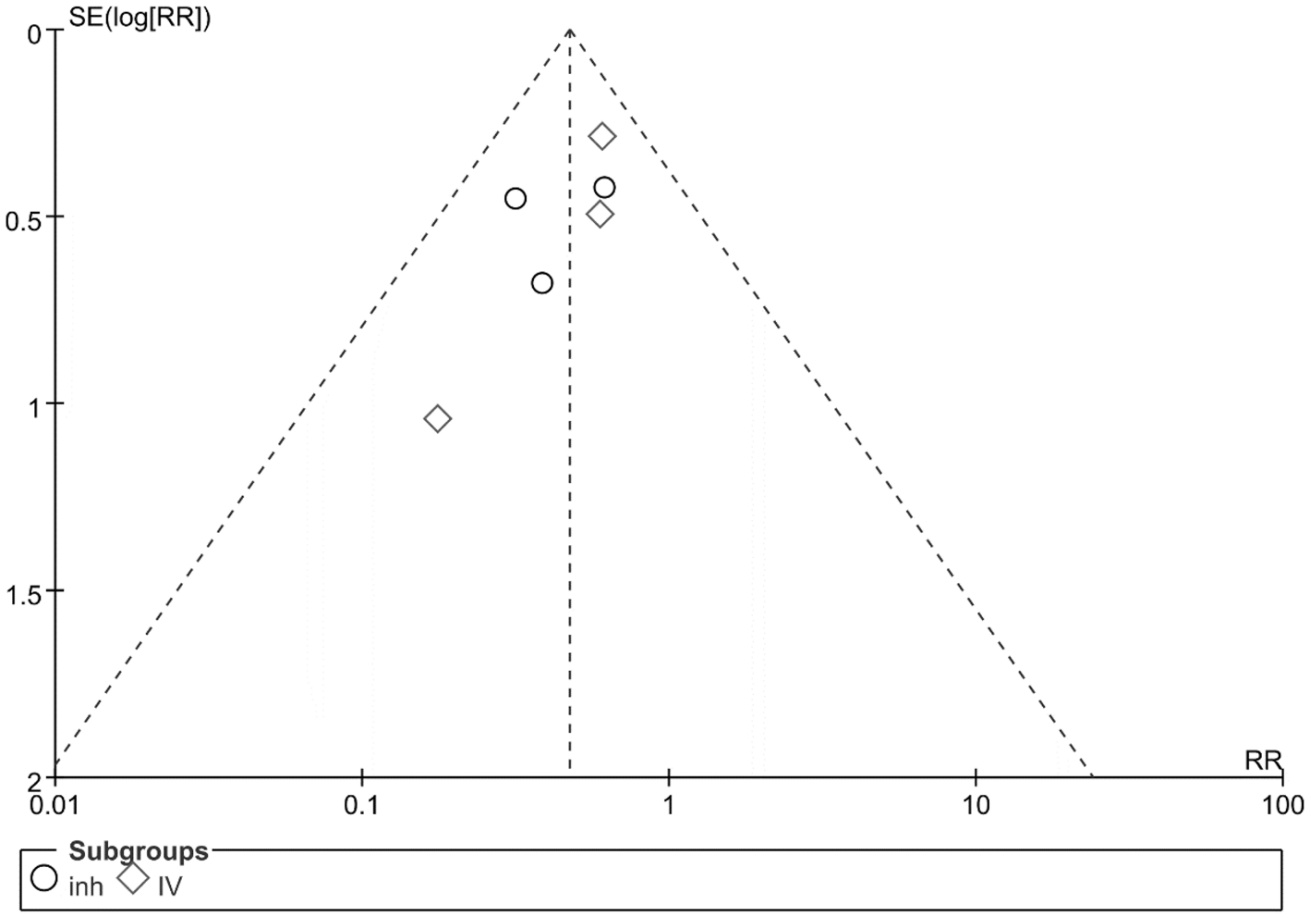
Funnel plot assessed for publication bias in the studies.

A visual inspection of the funnel plot indicated a symmetrical distribution, suggesting the absence of publication bias for Prophylactic use amphotericin B use in patients with hematologic disorders complicated by neutropenia (Fig. 6).

## Discussion

Fungal infection especially invasive fungal infection is one of the common complications of malignant hematological diseases, especially during the chemotherapy of acute myelocytic leukemia and acute lymphoblastic leukemia. The incidence of fungal infection in patients with hematological diseases ranges from 2 to 40% due to different chemotherapy intensities and durations of agranulocytosis^12,13^. Recently, patients with hematological diseases have been well treated by the large-scale application of immunosuppressants, glucocorticoids, broad-spectrum antibiotics, cytotoxic drugs and other therapeutic drugs. However, the incidence of deep fungal infections has also increased^14,15^. A new strategy of prophylactic antifungal therapy for susceptible people, for example, to reduce the incidence of mortality and fungal infection by prophylactic application of amphotericin B, has been proposed. The European guidelines recommend posaconazole as the first prophylactic agent for patients with blood disorders. Voriconazole, esaconazole and posaconazole are the first-line drugs for the treatment of invasive aspergillus disease, while Am B and esaconazole are the first-line drugs for the treatment of invasive mucormycosis^16^. However, the efficacy of prophylactic use in patients with hematologic disorders complicated by neutropenia is unclear. Herein, this study aims to comprehensively evaluate the indicators of this medication regimen through an evidence-based approach, providing a reference for clinical medication.

The meta-analysis showed that prophylactic application of Am B significantly reduced the fungal infection rate in patients with hematological disease complicated by neutropenia. The overall mortality of patients treated with Am B was slightly reduced compared with that of the control group (10.6% vs. 11.9%), but there was no significant difference among the groups. Thus, a subgroup analysis of overall mortality was conducted. The results showed that there was no significant difference between the two groups in drug-related mortality or infection-related mortality. Different infection forms could exist during the treatment of patients with hematological malignancies complicated by agranulocytosis, and fungal infections account for a relatively small proportion of total deaths^17^. In addition, two studies reported the incidence of colonization in patients, and the combined analysis showed that there was little difference in the incidence of colonization between the two groups due to the different body functions and immunosuppressive states of patients^9,10^. It is worth noting that guidelines from different countries and regions give some recommendations in the global application of antibiotics. The German Society of Hematology and Medical Oncology (DGHO) recommends that patients currently taking oral posaconazole or voriconazole prophylaxis should be switched to liposomal amphotericin B, recommended grade C-III, for patients with cancer with febrile neutropenia with pulmonary infiltration. In particular, amphotericin B liposomes (A-II) are recommended to be preferred in patients with suspected trichinosis^18^. Whereas the American College of Infectious Diseases recommends that for high-risk patients with chronic neutropenia and fever despite broad-spectrum antimicrobial therapy, antifungal agents of choice are amphotericin B-containing lipid formulations (strong recommendation, high quality evidence) and posaconazole suspension and voriconazole as one of the preferred drug choices for the prophylactic treatment of invasive pulmonary aspergillosis^19^. However, there is a lack of evidence from head-to-head high-quality studies to help us better compare their efficacy. In China, some guidelines also recommend the prophylactic use of amphotericin B in patients at high risk for invasive fungal disease^20^. The current global problem of antibiotic abuse is becoming increasingly serious, and it has been reported that the global consumption of antibiotics has increased by 46% since 2000^21^. The efficacy and safety of prophylactic antibiotic use is of greater concern, especially in high-risk populations, and we are not only concerned with process indicators, but also with the final outcomes, such as mortality, that different treatment modalities bring to patients. The results of this study showed that despite the advantage of amphotericin B in terms of fungal infection rates, it was not found to have a better effect on the primary outcome indicator of mortality. In addition, this study only included patients whose control group was placebo, and other studies involving drugs in prophylaxis were not included. We also hope that future studies will conduct in-depth studies and comparisons in terms of patient risk stratification (e.g., neutrophil levels), different drug dosage forms, dosing times, dosing regimens, and drug doses to help us make more refined drug adjustments and management in clinical practice.

This study included 6 high-quality controlled clinical studies with a sample size of 1019 cases after extensive and comprehensive retrieval. The number of studies and patients was small. And the risk of included studies’ bias is relatively high. There are still some limitations in this study: 1) The included studies are all overseas studies, which may have racial differences, restricting their applicability in China; 2) The included studies are too few to assess publication bias; 3) The baseline information of some studies is described incompletely; 4) There exist selection bias due to the languages of the included studies being limited to Chinese and English; 5) The strict inclusion and exclusion criteria of the included RCT studies restrict the extrapolation and applicability of the results.

In conclusion, compared with the control group, Am B can reduce the rate of fungal infection in patients with hematological disease complicated by neutropenia, but there is no significant advantage for the final outcome of patients. In addition, considering the possible adverse drug reactions and other problems associated with amphotericin B, in clinical practice, medical practitioners should fully evaluate the patient’s disease background and drug safety, follow the principle of individualized drug use, and use it with caution. In view of the limitations of this study, future studies carry out head-to-head high-quality randomized controlled studies from different mechanism drugs, in different dosage forms, in order to help us achieve refined management (Supplementary Information).

### Supplementary Information

Supplementary Information.

## Data Availability

The data that support the fundings of this study are available from the corresponding author upon reasonable request.
