# Supplementary material for: Prophylactic use amphotericin B use in patients with hematologic disorders complicated by neutropenia: a systematic review and meta-analysis
Source: Sci Rep. 2023 Aug 27;13:14008. doi: 10.1038/s41598-023-41268-1 (PMC10460793; doi:10.1038/s41598-023-41268-1)
Supplement: Supplementary file 1 — Supplementary Information. [file 41598_2023_41268_MOESM1_ESM.docx]

Appendix 1. Quality assessment of included studies

| study | Selection bias | | Performance bias | Detection bias | Attrition bias | Reporting bias |  |  |  |
| --- | --- | --- | --- | --- | --- | --- | --- | --- | --- |
|  | Random sequence generation | Allocation concealment | Blinding of participants and personnel | Blinding of outcome assessment | Incomplete outcome data | Selective reporting | Other bias | Total score (max =7) |  |
|  |  |  |  |  |  |  |  |  |  |
| Rijnders 2008^[6]^ | + | + | + | + | + | + | ? | 6 |  |
| Schwartz 1999^[7]^ | ? | + | - | - | + | + | ? | 3 |  |
| Behre 1995^[8]^ | ? | ? | - | - | + | + | ? | 2 |  |
| Riley 1994^[9]^ | ? | ? | + | + | + | + | ? | 4 |  |
| Perfect 1992^[10]^ | ? | ? | + | + | + | + | ? | 4 |  |
| Pizzo1982^[11]^ | ? | ? | + | + | + | + | ? | 4 |  |
